# Intraprocedural echocardiographic guidance in transcatheter aortic valve implantation

**DOI:** 10.1186/s44348-026-00094-5

**Published:** 2026-09-01

**Authors:** Tai Meng Chen, Mohammed Rizwan Amanullah, See Hooi Ewe

**Affiliations:** 1 Department of Cardiology, National Heart Centre Singapore, Singapore, Singapore; 2 Department of Cardiology, Hospital Raja Permaisuri Bainun, Ipoh, Malaysia

**Keywords:** Aortic Valve Stenosis, Transcatheter Aortic Valve Replacement, Echocardiography

## Abstract

Transcatheter aortic valve implantation (TAVI) is an established treatment for severe aortic stenosis across all surgical risk groups. Traditionally performed under general anesthesia with transesophageal echocardiography (TEE) guidance, TAVI has evolved toward a minimalist approach using local anesthesia and conscious sedation (LACS), supported by transthoracic echocardiography (TTE) or fluoroscopy alone. Despite this shift, intraprocedural imaging remains valuable as it offers real-time imaging guidance throughout the procedures, allowing for rapid and accurate assessment of complication and procedural results. Echocardiographic modalities used during TAVI include TTE, TEE (adult-, mini-, or micro-TEE) and intracardiac echocardiography (ICE), each with its own advantages and limitations. While TTE is widely used in uncomplicated cases, its image quality may be suboptimal, leading to missing complications and underestimation of paravalvular leak (PVL). In contrast, TEE provides superior spatial resolution, enabling comprehensive real-time assessment and improved PVL detection and other complications. Moreover, 3D TEE offers accurate assessment of the aortic annulus and aids in sizing of the prosthesis when computed tomography is suboptimal or unavailable. ICE is an attractive alternative as it provides real-time high-resolution images and can be performed under LACS by the same operator, although it is costly with single use and requires additional venous access. ICE may be particularly helpful in patients with challenging TTE windows, or in those with contraindications for TEE. We propose an upfront intraprocedural TEE strategy in patients with high-risk anatomies undergoing TAVI, and those with poor TTE window or renal impairment in whom minimal contrast usage is preferred. Mini-TEE (with a smaller probe) is the preferred modality for intraprocedural guidance, which has 3D imaging with good resolution, and is well tolerated under LACS. In conclusion, imaging strategies in TAVI should be individualized. While minimalist approaches are feasible, TEE remains indispensable in complex cases to optimize outcomes and minimize complications.

## Background

Transcatheter aortic valve implantation (TAVI) has become an established minimally invasive treatment for patients with severe aortic stenosis (AS) across the entire spectrum of surgical risk, including those at high, intermediate, and low risk. Clinical outcomes have been shown to be comparable or superior to those of surgical aortic valve (AV) replacement [1–4]. Conventionally, TAVI is performed under general anesthesia (GA) with intraprocedural transesophageal echocardiography (TEE) guidance to evaluate aortic root anatomy and facilitate valve positioning [5]. Most cases are now performed via the transfemoral approach, reflective of growing operator experience, advancements in valve design, and improvements in delivery systems. With improved procedural efficiency and shortened operative times, TAVI has evolved from a procedure done routinely under GA to one that can be safely performed with local anesthesia and conscious sedation (LACS). Available evidence indicates that this minimalist approach is less invasive, promotes earlier ambulation and discharge, and does not compromise short term clinical outcomes [6, 7]. Accordingly, with the increased use of LACS, there is reduced reliance on TEE during TAVI as prolonged TEE use may be uncomfortable in conscious patients. However, echocardiographic imaging during TAVI procedure remains critical to ensure proper valve deployment and functioning, allowing for rapid detection of complication and prompt initiation of appropriate intervention.

This review therefore examines the role of echocardiographic imaging in TAVI for native AS, outlining the various echocardiography modalities available, and their appropriate use during the procedure, including key advantages and limitations of each modality. Finally, careful patient selection remains fundamental to achieving best optimal procedural outcomes while anticipating potential complications.

## Echocardiographic modalities for guidance during TAVI

Echocardiographic guidance during TAVI procedure has evolved progressively from the initial TEE guidance (under GA) to a more efficient and minimalist approach performed under LACS, supported by focused transthoracic echocardiography (TTE) imaging done before and immediately following valve deployment. In some high volume and experienced centers, TAVI has been performed entirely under fluoroscopy without echocardiographic support, albeit more contrast aortography [8]. The echocardiography modalities use during TAVI can be broadly categorized into TTE and TEE imaging. TEE may be further classified according to the size of the commercially available probes, including the standard adult-TEE, the smaller mini-TEE, and the smallest micro-TEE probes. Both the adult- and mini-TEE probes can support 3D imaging. Recent years have seen an emergence of intracardiac echocardiography (ICE) imaging use in TAVI. Table 1 summarizes the important differences between the various echocardiography modalities and their distinct characteristics.

**Table 1:** Comparison between various echocardiography modalities use in TAVI guidance and their respective advantages and limitations

	TTE	Adult-TEE	Mini-TEE		Micro-TEE	ICE
Probe head width or catheter size	-	14–17 mm	11 mm		8 mm	8–10F catheter (2.7–3.3 mm in diameter)
Procedure invasiveness	Noninvasive	Semi-invasive	Less invasive compared to adult-TEE			Invasive (femoral or jugular access)
Contemporary anesthesia requirement	LACS	GA	LACS			LACS
Imaging and Doppler modes	2D, 3D, biplane, PW Doppler, CW Doppler, and color (2D, 3D)	2D, 3D, biplane, PW Doppler, CW Doppler, and color (2D, 3D)	2D, 3D, biplane, PW Doppler, CW Doppler, and color (2D, 3D)	2D, PW Doppler, CW Doppler, color (2D only) No 3D or biplane imaging		2D, PW Doppler, CW Doppler, and color (for 2D ICE) Additional 3D, biplane and color (for 3D ICE)
Imaging advantage	Noninvasive transthoracic imaging	Superior 2D and 3D image resolution	Comparable 2D but lower 3D image resolution compared to adult-TEE	Lower 2D resolution than adult- and mini-TEE		Superior 2D and 3D image resolution; preferred when TEE is contraindicated (due to esophageal stricture, ulceration, or varices) or when conscious sedation is preferred in patients with poor TTE windows
Imaging disadvantage	Imaging is less reliable; dependent on acoustic windows, body habitus, chest/lung condition and positioning of patient	Patient discomfort with risk of injury to the oropharynx or esophagus; longer procedure time under GA	Minimal patient discomfort with lower risk of injury to the oropharynx or esophagus compared to adult-TEE; shorter procedure time as can be performed under conscious sedation			Requires additional venous access with risk of vascular injury, pericardial effusion and interference with pacing wires; transient atrial or ventricular arrhythmias; narrow field of view compared to TEE; single use and higher cost; learning curve
Disruption of surgical field sterility	Potential disruption but can be minimized with the use of sterile TTE probe covers	No disruption				No disruption
Concurrent imaging with fluoroscopy	No	Yes, useful for intraprocedural guidance and monitoring; minor adjustment to probe position is required to avoid interference with fluoroscopy during valve deployment				Yes, useful for intraprocedural guidance and monitoring; minor adjustment to catheter position is required to avoid interference with fluoroscopy during valve deployment
Continuous imaging and procedural monitoring	Intermittent imaging or monitoring when required; slight delay in image acquisition	Provides continuous intraprocedural imaging and real-time assessment and prompt detection of complication				Provides continuous intraprocedural imaging and real-time assessment and prompt detection of complication
Assessment of aortic root anatomy and annular sizing	No, poor resolution	Yes, 3D capability and adequate image resolution		No, lack of 3D capability		Yes,3D ICE catheters have 3D capability although limited by narrow field of view compared to 3D TEE

CW, continuous wave; GA, general anesthesia; ICE, intracardiac echocardiography; LACS, local anesthesia and conscious sedation; PW, pulsed wave; TAVI, transcatheter aortic valve implantation; TEE, transesophageal echocardiography; TTE, transthoracic echocardiography

## Contemporary role of intraprocedural imaging in TAVI procedures

Imaging cardiologist is an integral part of the “heart team” and plays a crucial role in TAVI preprocedural planning and imaging. While TTE is frequently utilized for guidance during uncomplicated TAVI procedures performed under conscious sedation [9], its utility for intraprocedural imaging may be limited (Table 1). Anatomical factors such as imaging in the supine rather than left lateral position, hyperinflated lungs, chest wall deformity and body habitus can significantly compromise image quality. In such situation with suboptimal windows, important findings such as paravalvular leak (PVL), concomitant valvular pathology, intracardiac thrombus, and pericardial effusion may be missed. In this regard, minimalist TAVI with TTE guidance has been shown to be associated with a higher incidence of additional maneuvers to reduce PVL such as balloon following dilatation (38% vs. 17%, P < 0.001) and second valve deployment (7% vs. 2%, P = 0.026) when compared to TEE-guided TAVI procedures [10].

**Fig. 1:**
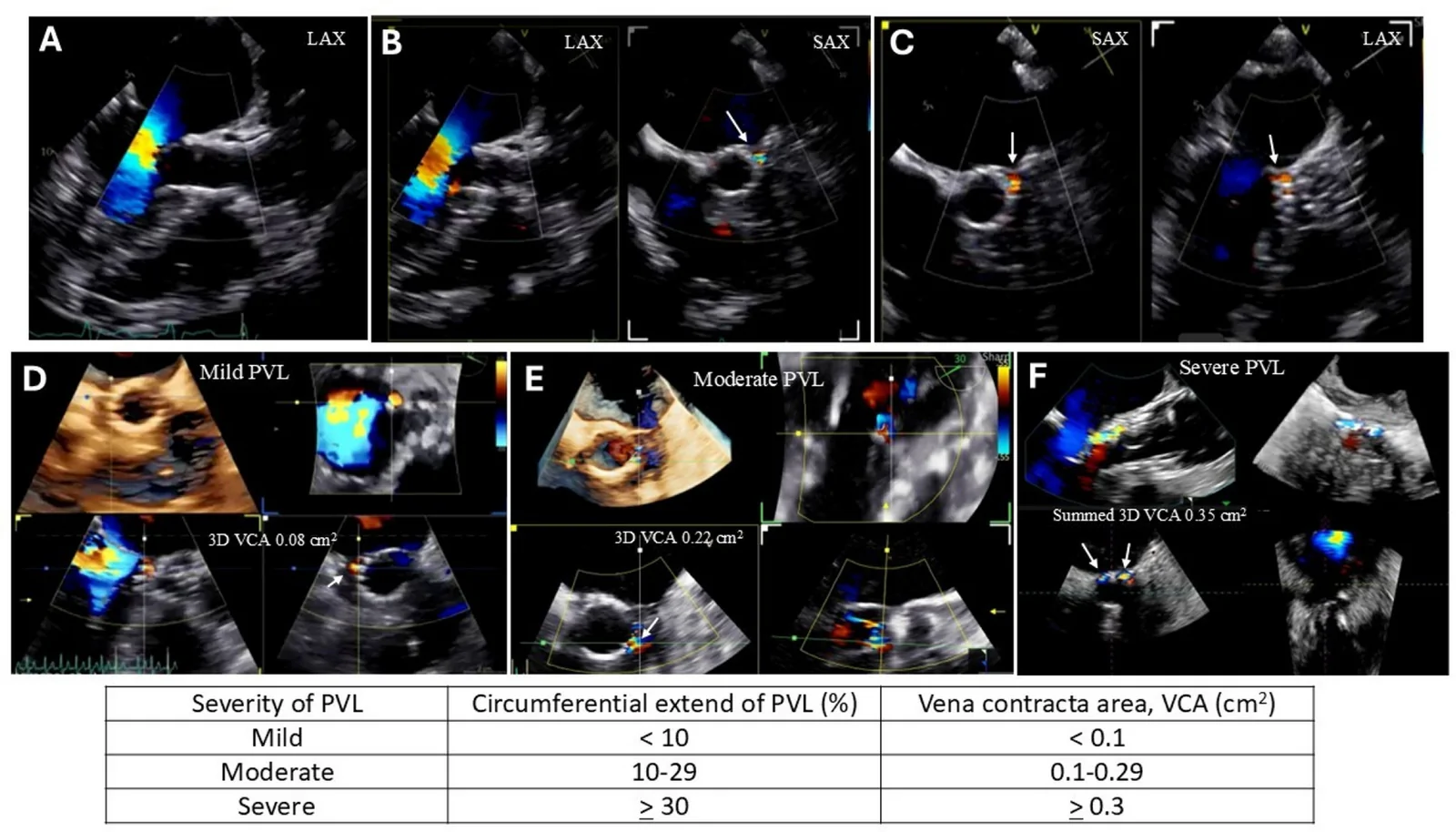
Assessment of paravalvular aortic regurgitation on transesophageal echocardiography. **A** Initial mid-esophageal long-axis (LAX) view showing no paravalvular leak (PVL), simultaneous biplane imaging with cut-plane at the level of inflow edge of the transcatheter valve to locate the PVL, at 2 o’clock of **B** the corresponding short-axis (SAX) view. **C** Further rotation of the probe confirming the eccentric PVL jet, in the off-axis mid-esophageal LAX view. Severity of PVL is graded by its circumferential extent: less than 10% is considered mild, 10% to 29% is moderate, and greater than 30% is severe; or based on the planimetered 3D vena contracta area (VCA) of the PVL jets. (D–F) For multiple PVL jets, summation of all their respective 3D VCA may be employed

**Fig. 2:**
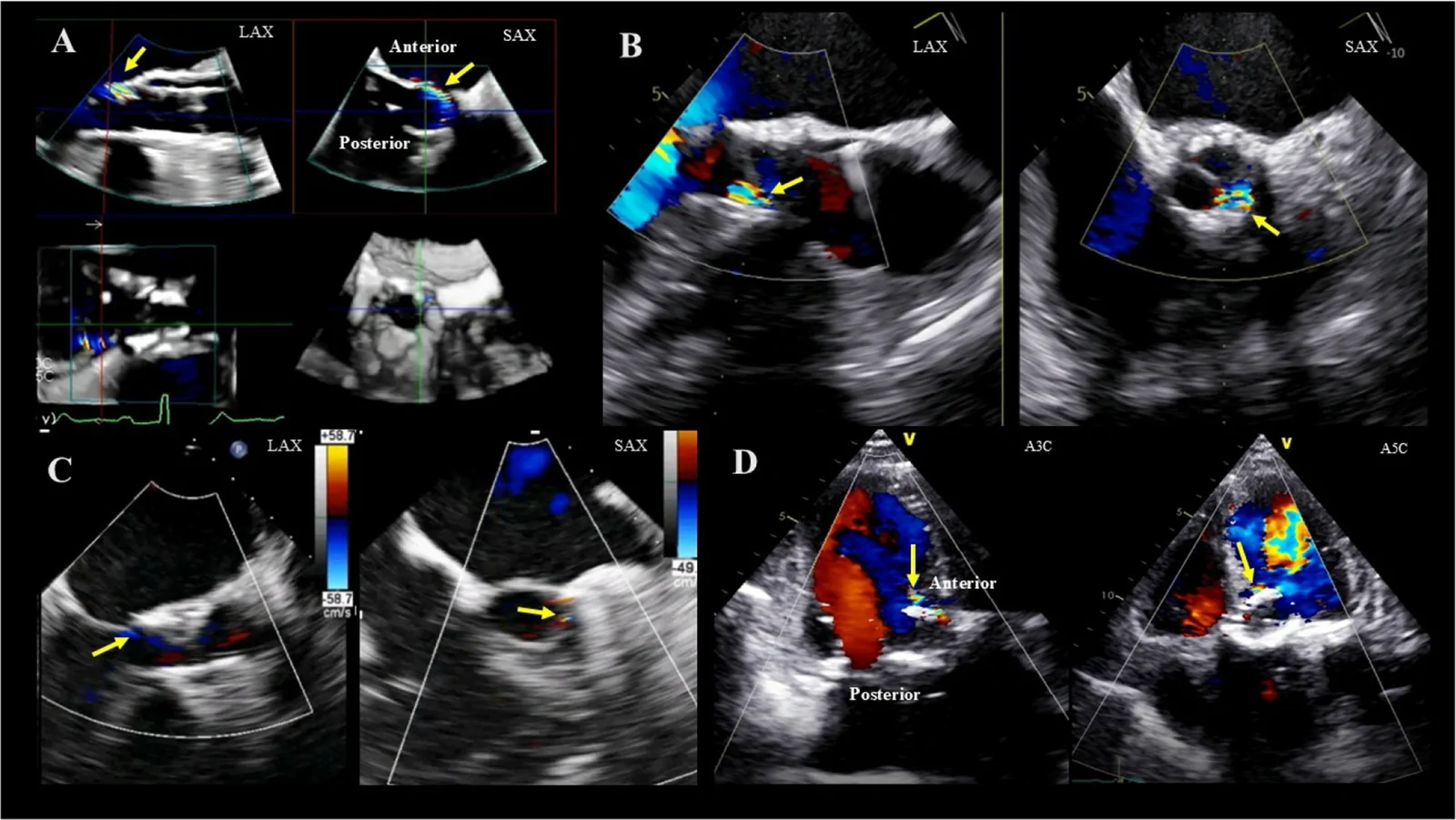
Paravalvular and transvalvular aortic regurgitation (AR) on echocardiography. **A** A posteriorly located paravalvular AR jet is best visualized in the mid-esophageal long-axis (LAX) view on transesophageal echocardiography (TEE) images acquired using the standard adult-TEE probe. Based on the short-axis (SAX) view in 3D TEE, the severity of the paravalvular AR is graded as moderate, involving 20% of its circumferential extent. **B** Transvalvular AR jet confirmed on the corresponding short-axis view, using biplane imaging in the mid-esophageal LAX view on mini-TEE. **C** Micro-TEE detected mild paravalvular leak (PVL) in the LAX view, confirmed on the SAX views, at 2 o’clock. **D** An anteriorly located paravalvular AR is best visualized on the transthoracic echocardiography, in the apical three- or five-chamber view. Due to the acoustic shadowing of the prosthetic valve, it is important to tilt the ultrasound probe in multiple directions (upward or sideways) and in unconventional planes to detect and localize the paravalvular AR. Arrows indicate PVL

Moreover, Zaid et al. [11] reported that 91 of 475 patients (19%) who underwent TTE-guided minimalist TAVI procedures had mild or more PVL at predischarge TTE. Forty-two patients (46%) of these PVL were missed by intraprocedural TTE. Importantly, missed PVL rate was significantly higher for posteriorly located jets when compared to anteriorly located jets in both the parasternal long-axis (63% vs. 25%, P = 0.005) and parasternal short-axis (67% vs. 24%, P = 0.001) views but not apical three-chamber view (59% vs. 40%, P = 0.28). Therefore, when TTE is used, it is imperative to tilt the ultrasound probe in multiple directions (upward or sideways) using multiwindow approach to detect and localize the PVL [12]. In contrast, TEE provides superior spatial resolution and better at detection of posteriorly located jets (close to transducer), while anteriorly located jets may be shadowed with TEE (further from transducer). Nonetheless, this limitation can be overcome by multilevel and simultaneous biplane imaging at mid-esophageal long-axis view (with corresponding orthogonal short-axis view), sweeping along just below the level of the valve within the left ventricular outflow tract (LVOT), and deep transgastric views for comprehensive intraprocedural assessment of the PVL [13]. Figure 1 shows the recommended method for PVL assessment and grading of its severity. It is imperative to identify and differentiate this from transvalvular aortic regurgitation (AR). Figure 2 shows examples of paravalvular and transvalvular AR on echocardiography. Any degree of PVL after TAVI is associated with worse clinical outcomes and attempts should be aimed at mitigating or elimination of the PVL during the index TAVI procedure in the Cath lab [14].

Although TAVI is considered a generally safe procedure, the recent 2025 European Society of Cardiology Guidelines on valvular heart disease define higher risk TAVI candidates as those with low coronary ostia, difficult femoral access, bicuspid AV, extensive calcification extending into the LVOT, severe left ventricular (LV) and/or right ventricular (RV) dysfunction, isolated AV regurgitation, multivalvular involvement, complex coronary artery disease and severe extracardiac disease including renal dysfunction and/or pulmonary hypertension [15]. As these cases are more complex and are associated with higher complication rates, they should be performed in high volume, experienced centers with better infrastructural and “heart team” support. In such settings, not only is preprocedural planning crucial, prompt and accurate intraprocedural imaging is essential to guide and monitor these higher risk procedures. In this regard, intraprocedural TEE remains the primary imaging modality of choice as it permits continuous, real-time imaging to assess cardiac structure and prosthetic valve function, for rapid detection of complications alongside fluoroscopy.

Chronic kidney disease and end-stage renal disease are prevalent among TAVI candidates, affecting 33.5% and 4.1% of patients, respectively [16, 17]. The standard TAVI workflow, including preprocedural computed tomography (CT) for planning and intraprocedural angiography for valve positioning and postprocedural vascular assessment, relies heavily on iodinated contrast, which elevates the risk of acute kidney injury in patients with renal dysfunction and elderly patients at risk of acute kidney injury. To mitigate this risk, TAVI can be performed using low or zero contrast protocols guided by intraprocedural TEE [18]. Intraprocedural TEE may help reduce contrast use in patients with underlying renal impairment, by guiding valve positioning and deployment, as well as in AR assessment post TAVI without the need for contrast aortography.

**Fig. 3:**
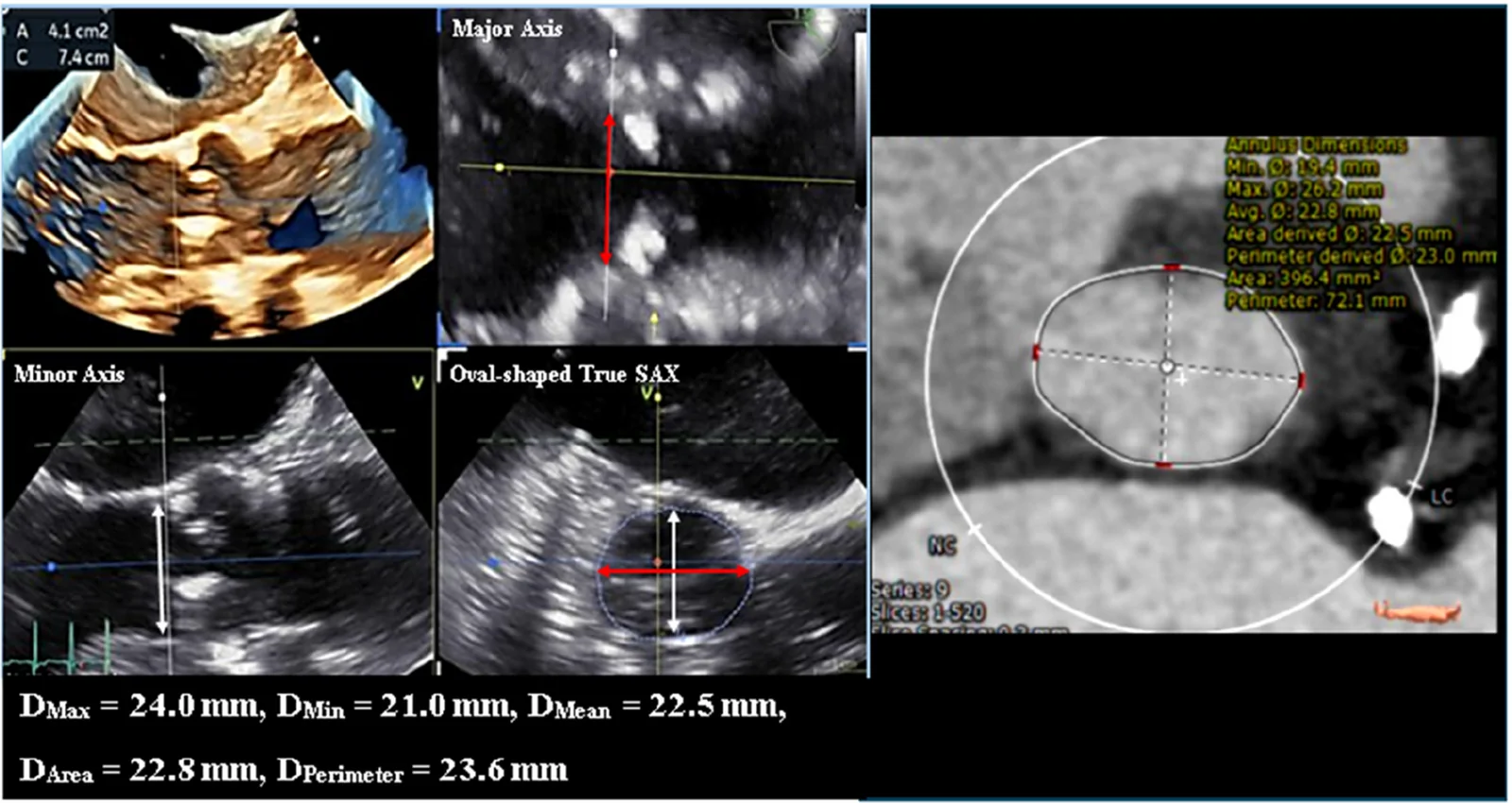
The 3D multiplanar imaging for the aortic annulus on transesophageal echocardiography (TEE). Left panel shows annular dimension obtained on 3D TEE via direct planimetry method using user-defined multiplanar approach, whereby the annular plane is a virtual plane at the level of the lowest attachment site of the three aortic cusps. As the aortic annulus is oval in shape, the long-axis plane diameter (from 2D TEE) typical represents its minor dimension (white arrow), while its maximal dimension is derived from the coronal plane obtained using multiplanar reconstruction of the acquired 3D volume dataset (red arrow). From 3D TEE, the annular area and perimeter were 410 mm^2^ and 74 mm, respectively. The right panel shows a computed tomography–derived aortic annular dimensions in the same patient. SAX, short axis

**Fig. 4:**
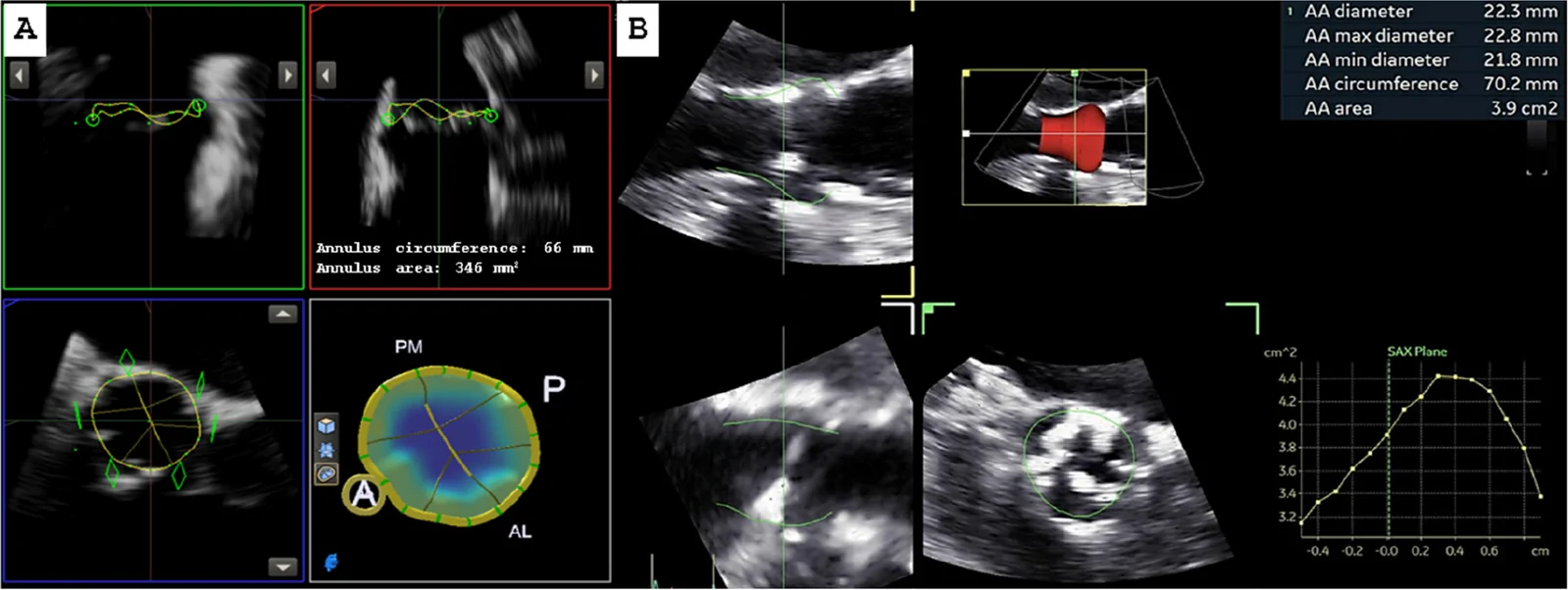
Indirect planimetry and semi-automated measurements of the aortic annulus on transesophageal echocardiography. **A** An example of indirect planimetry using mitral valve quantification software that uses the orthogonal long-axis view to identify the annular plane in its corresponding short-axis view, followed by provision of its planimetered area and circumference without hand-tracing. **B** New semiautomated aortic valve quantification software is available on cart that can automatically provide the annular dimension in terms of its diameter (maximal, minimal and averaged), area and circumference, after verification of the annular plane on 3D multiplanar reconstruction

**Fig. 5:**
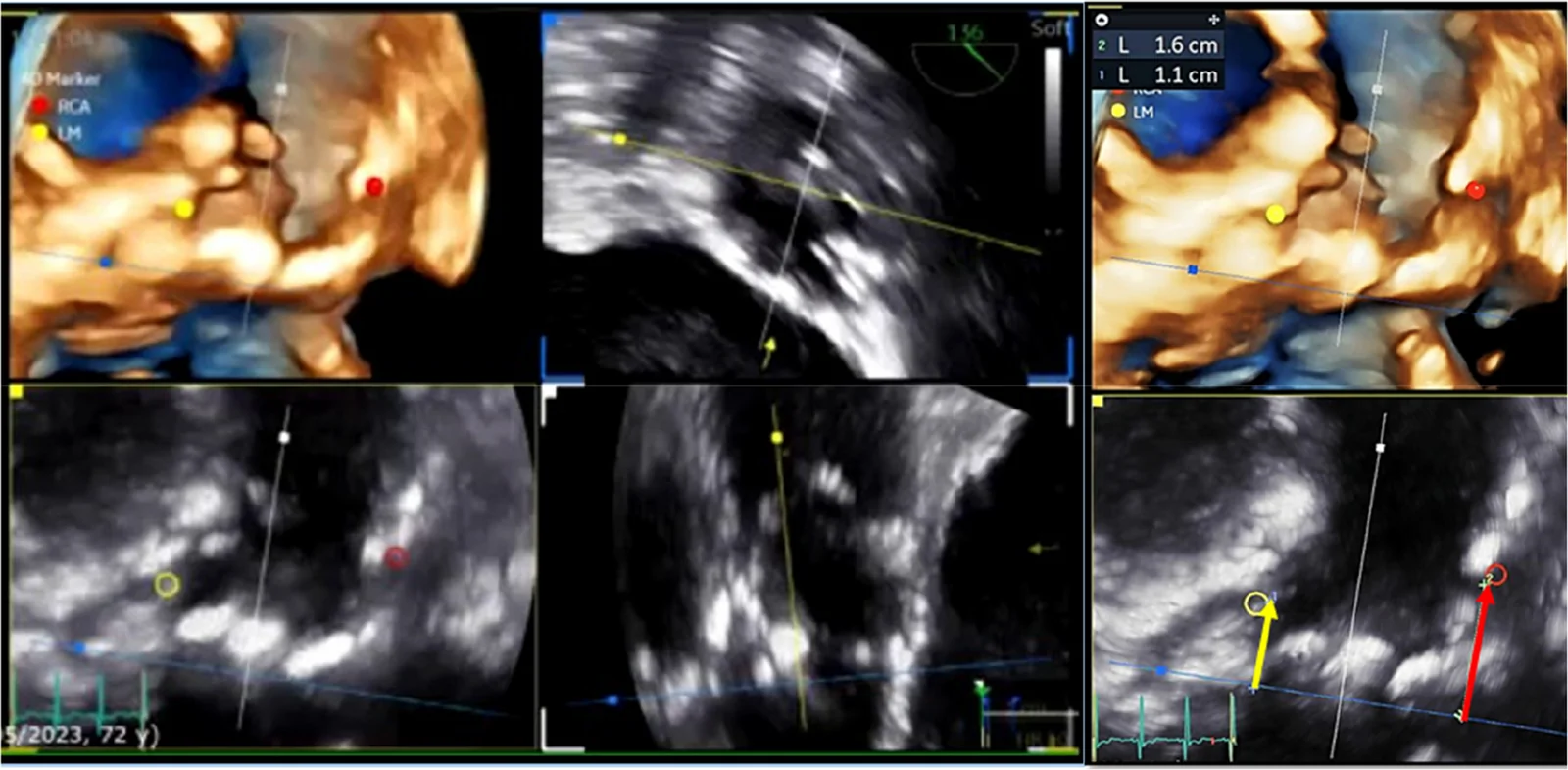
Multiplanar imaging for coronary height measurements with aid of markers on 3D transesophageal echocardiography (TEE). Left and right coronary artery ostia are marked as the yellow and red circles, respectively on 3D TEE dataset. Using 3D multiplanar reconstruction and the markers, the height of both coronary artery ostia are easily identified on the volume rendering view. In this case, the left coronary ostium is measured at 11 mm, while that of the right coronary ostium is 16 mm above the annular plane, respectively

For example, 3D TEE either performed at the time of TAVI or prior to TAVI, offers an alternative imaging to evaluate the aortic root when iodinated contrast use is prohibited. Good correlation between 3D TEE and CT measurements have been published using either the direct planimetry method with 3D multiplanar reconstruction (MPR) or the semiautomated software for assessment of the aortic annular dimension (Figs. 3, 4), including measurement of the coronary heights (Fig. 5), although 3D TEE routinely underestimate the annular measurement by 4% to 7% when compared to CT but does not significantly alter the transcatheter sizing [19]. Additionally, intraprocedural 3D TEE helps to confirm the aortic root and annular measurements prior to interprocedurally in cases of borderline valve sizing or when CT image quality is suboptimal [20–24].

Most importantly, intraprocedural TEE allows for close monitoring and enables immediate detection of complications in challenging anatomy that are often associated with higher complication rates. In light of these considerations, we would propose an upfront intraprocedural TEE strategy during TAVI in patients with high-risk anatomy, as well as those with poor TTE window or renal impairment in whom minimal contrast usage is preferred (Table 2). To balance the need of continuous imaging and patient comfort, the use of mini-TEE probe (instead of adult-TEE probe) with a smaller footprint is a preferred modality, that is capable of 3D imaging with equivalent imaging quality and can be safely performed under LACS [25].

**Table 2:** Situations where upfront TEE is preferred over TTE during TAVI

	Clinical indication
Tolerability	Comorbidities precluding the ability to protect airway (such as severe lung disease or obstructive sleep apnea) during TAVI procedure, necessitating conversion to GA
Imaging	Borderline preprocedural aortic annular and root measurements on CT that requires confirmation Poor TTE window
High-risk anatomy	Bicuspid aortic valve: risk of PVL and valve embolization Extensive leaflet or LVOT calcification: risk of PVL or valve migration Low coronary ostia: risk of coronary obstruction Small hypertrophic LV: risk of guidewire injuring mitral valve apparatus Severe basal septal hypertrophy: risk of LVOT obstruction and valve embolization Severe LV and/or RV impairment: risk of cardiac arrest or hypotension Challenging femoral anatomy: risk of vascular injury and bleeding or unsuccessful access that requires conversion to nonfemoral access
Chronic kidney disease	Risk of acute kidney injury, for minimal contrast usage

CT, computed tomography; GA, General anesthesia; LV, left ventricular; LVOT, left ventricular outflow tract; PVL, paravalvular leak; RV, right ventricular; TAVI, transcatheter aortic valve implantation; TEE, transesophageal echocardiography; TTE, transthoracic echocardiography

## Intraprocedural echocardiography during TAVI

Table 3 summarizes the key intraprocedural imaging steps and the imaging assessment provided by various echocardiography modalities during these steps.

**Table 3:** Key TAVI intraprocedural imaging steps and imaging assessment by various echocardiography modalities

Procedural step	2D/3D TEE imaging	TTE (2D and biplane)	2D/3D ICE imaging
Baseline assessment (pre-TAVI imaging)	Ventricular function and assessment of all valves function and severity Document any pericardial effusion at baseline Confirm the aortic root and annular dimension in cases of borderline valve sizing or when CT image quality is suboptimal (only with 3D TEE imaging)	PLAX, PSAX, and apical views	Longitudinal view from cavoatrial junction Transventricular long axis view of LV (from ICE catheter in RV)
Pacing wire and stiff wire position	Confirm pacing wire position in RV To guide valve crossing (in challenging anatomy such as bicuspid or heavily calcified valve) Exclude interference with mitral valve apparatus and pericardial effusion	Limited but possible in PLAX and apical views	Longitudinal view from cavoatrial junction
Balloon valvuloplasty	Evaluate for AR post balloon valvuloplasty Exclude aortic root injury and determine the cause of hypotension during or post balloon valvuloplasty such as acute AR or cardiac stunting post rapid pacing	Limited but possible in PLAX, PSAX, and apical views	Longitudinal view from cavoatrial junction
Positioning of THV	For self-expanding valve: Confirm the proximal edge is ≈5 mm below the annulus and look for paravalvular AR prior to full deployment (for the retrievable and repositionable platform such as the CoreValve [Medtronic] or Portico/Navitor valves [Abbott]) For second generation valve: confirm the proximal part is ≈5 mm below the annulus with optimal final position at ≈2 mm below the annulus For third generation balloon-expandable valve: ensure distal part is below the STJ, covering the native leaflets	Limited but possible in PLAX view	Longitudinal view from cavoatrial junction
Post-deployment	Assess for prosthetic valve positioning, shape and leaflet motion Detect paravalvular AR (focusing on the LVOT and inflow edge of the prosthetic valve) and determine the mechanism of AR Assess coronary artery patency, ventricular and mitral valve function Exclude complications such as pericardial effusion, perforation cardiac tamponade, aortic dissection and hypovolaemia from major bleeding	PLAX, PSAX, and apical views; however, detection of complications may be delayed due to intermittent imaging and the need to interrupt fluoroscopy and sterile field	Longitudinal and short-axis views from cavoatrial junction Transventricular long-axis view of LV (from ICE catheter in RV)

AR, aortic regurgitation; CT, computed tomography; ICE, intracardiac echocardiography; LV, left ventricle; LVOT, left ventricular outflow tract; PLAX, parasternal long-axis; PSAX, parasternal short-axis; RV, right ventricle; STJ, sinotubular junction; TAVI, transcatheter aortic valve implantation; TEE; transesophageal echocardiography; THV, transcatheter heart valve; TTE, transthoracic echocardiography

### Baseline pre-TAVI imaging

For intraprocedural imaging prior to TAVI deployment, either TTE or TEE may be utilized to establish a baseline morphological and hemodynamic assessment. At minimum, this evaluation should include full assessment of biventricular size and function, concomitant valvular lesion and presence of any pericardial effusion. In addition, the baseline mitral regurgitation (MR) should be documented, as changes in the severity of MR during the procedure may indicate mechanical interference with the mitral apparatus from manipulation of the catheter, wire or delivery system. In cases of borderline valve sizing or when preprocedural CT image quality is suboptimal, a 3D capable TEE is recommended to fully evaluate the AV complex (valve anatomy, severity and location of calcification) and aortic root to aid in device sizing and estimation of PVL risk based on patient anatomy. Of note, it is important to highlight that 2D TTE or 2D TEE alone is not recommended for annular sizing as it typically yields smaller annular diameters when compared to 3D TEE or CT [20–24], highlighting the importance of 3D derived annular sizing. As the aortic annulus is oval in shape, the long-axis plane diameter (from 2D TTE or TEE) typical represents its shorter dimension, while its maximal dimension is derived from the coronal plane obtained using MPR of the acquired 3D volume dataset (Fig. 3). Therefore, it is currently recommended to size the transcatheter heart valve (THV) either by the area or perimeter derived method during systole, for balloon-expandable or self-expanding TAVI valve, respectively. To facilitate this workflow, commercially available semi-automatic software is available to provide an automated reconstruction of the aortic annulus via indirect or direct methods on 3D TEE (Fig. 4). Moreover, 3D TEE imaging allows for measurement of the annulus-to-left main distance (from the coronal plane using MPR) which are comparable to CT [26]. to assess the risk of coronary occlusion during TAVI (Fig. 5). It is generally considered safe when the coronary artery height is at least 10 mm from the annular plane.

### During valve implantation

**Fig. 6:**
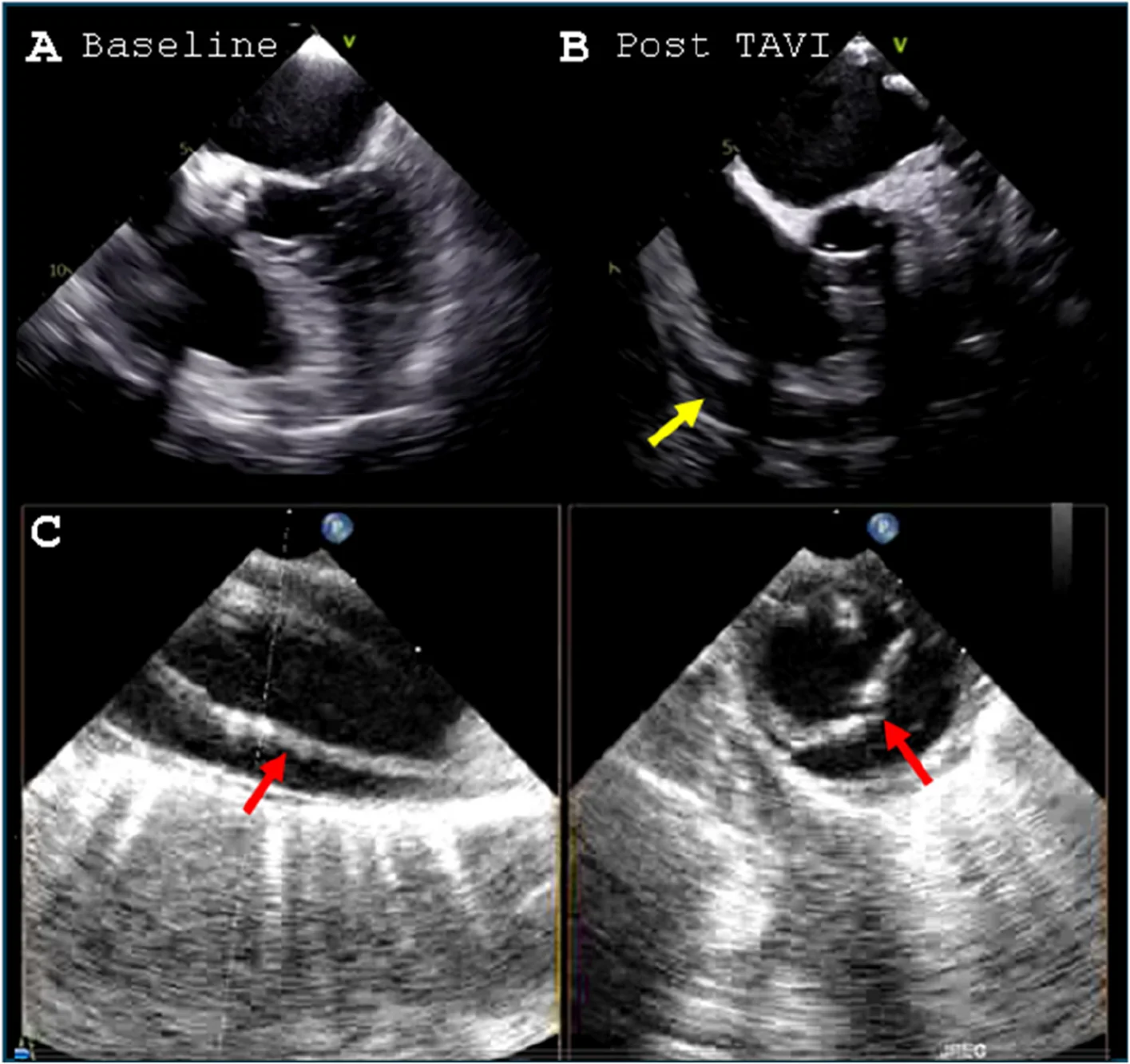
Potentially life-threatening complications detected by intraprocedural transesophageal echocardiography (TEE) during transcatheter aortic valve implantation (TAVI) procedure. **A** Pre-TAVI TEE at baseline showing minimal pericardial effusion. **B** Post-TAVI TEE showing a moderate size pericardial effusion (yellow arrow) caused by right ventricular perforation from pacing wire, that was promptly identified and timely drained without any hemodynamic compromise. **C** Aortic dissection demonstrated on biplane TEE imaging in a patient who developed hypotension. Dissection flap was visualized in the descending aorta (red arrow)

**Fig. 7:**
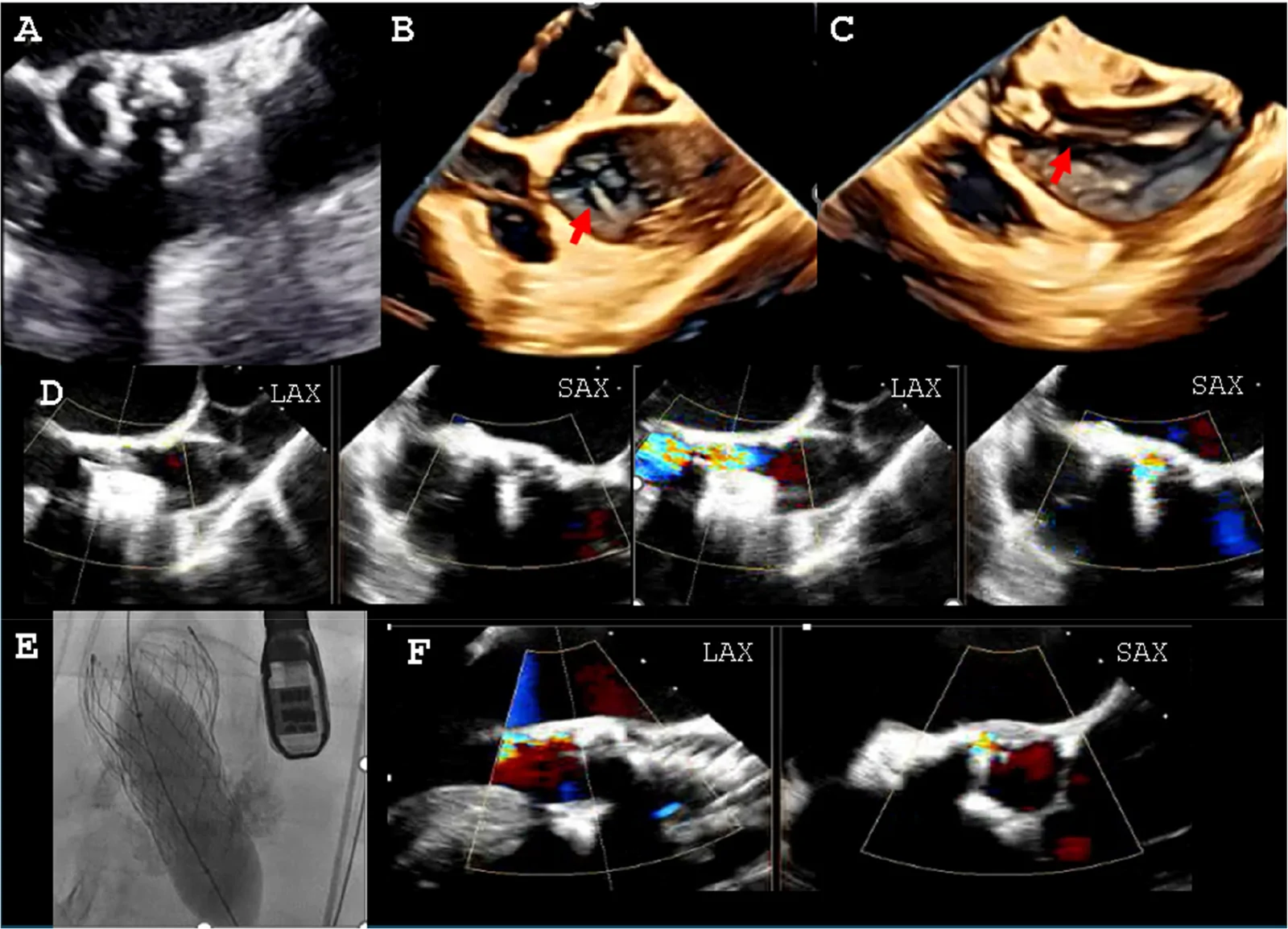
Transcatheter aortic valve implantation (TAVI) in bicuspid aortic valve. **A** Real-time guidance using mini-transesophageal echocardiography (TEE) is highly valuable in challenging valve anatomy such as bicuspid AV with severe calcification. Under direct 3D TEE guidance, using (volume rendered) (**B**) *en face* and (**C**) lateral views of the aortic valve (AV), the guidewire (arrow) is directed towards the narrowed valve orifice that helps in visualizing wire crossing (arrow) of the severely calcified bicuspid AV. **D** A case of underexpanded transcatheter heart valve in a type 0 bicuspid AV with severe paravalvular leak (PVL) post TAVI on adult-TEE imaging, **E** which requires reballooning under fluoroscopy guidance. **F** Final residual mild PVL with circularization of the CoreValve (Medtronic) at the annular level. LAX, long axis; SAX, short axis

**Fig. 8:**
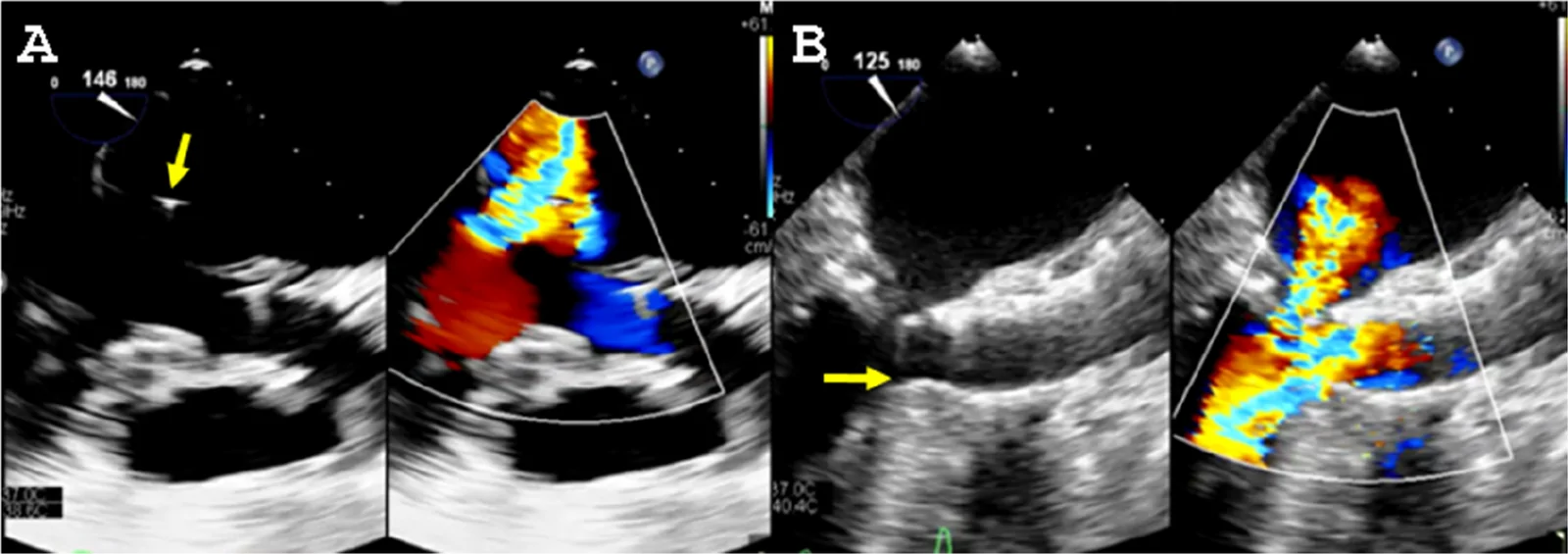
Detection of new or worsening mitral regurgitation (MR) during transcatheter aortic valve implantation (TAVI) procedure. **A** Significant MR jet due to guidewire (arrow) interfering with subvalvular apparatus and mitral leaflet motion. **B** Systolic anterior motion of anterior mitral leaflet giving rise to MR and dynamic left ventricular outflow tract obstruction post TAVI due to thick basal (sigmoid) septum (arrow) confirmed on intraprocedural transesophageal echocardiography imaging

Rapid pacing is essential for majority of the TAVI procedures to reduce cardiac output temporarily for precise valve position and deployment. This is traditionally performed via a temporary transvenous lead placement in the RV apex, although direct LV pacing via the stiff wire in the LV are becoming more common [27]. Although lead-related RV perforation resulting in cardiac tamponade is not common, confirmation of the pacing lead position in RV apex and early detection of pericardial effusion accumulation is the recommended first step (Fig. 6). Next, navigating a guidewire retrogradely through a stenotic AV can be challenging, particularly in patients with severely calcified leaflets or bicuspid valves with asymmetrical valve orifice. This step can be imaged on TEE, which not only provides detailed assessment of the AV morphology and calcification, but also real-time visualizing of the wire location throughout the procedure (Fig. 7). In addition, both 2D and 3D TEE permits clear visualization of the retrograde stiff wire, ensuring the coiled tip is safely positioned at the LV apex for stability while preventing entanglement or injury to the mitral apparatus, which could exacerbate MR (Fig. 8).

**Fig. 9:**
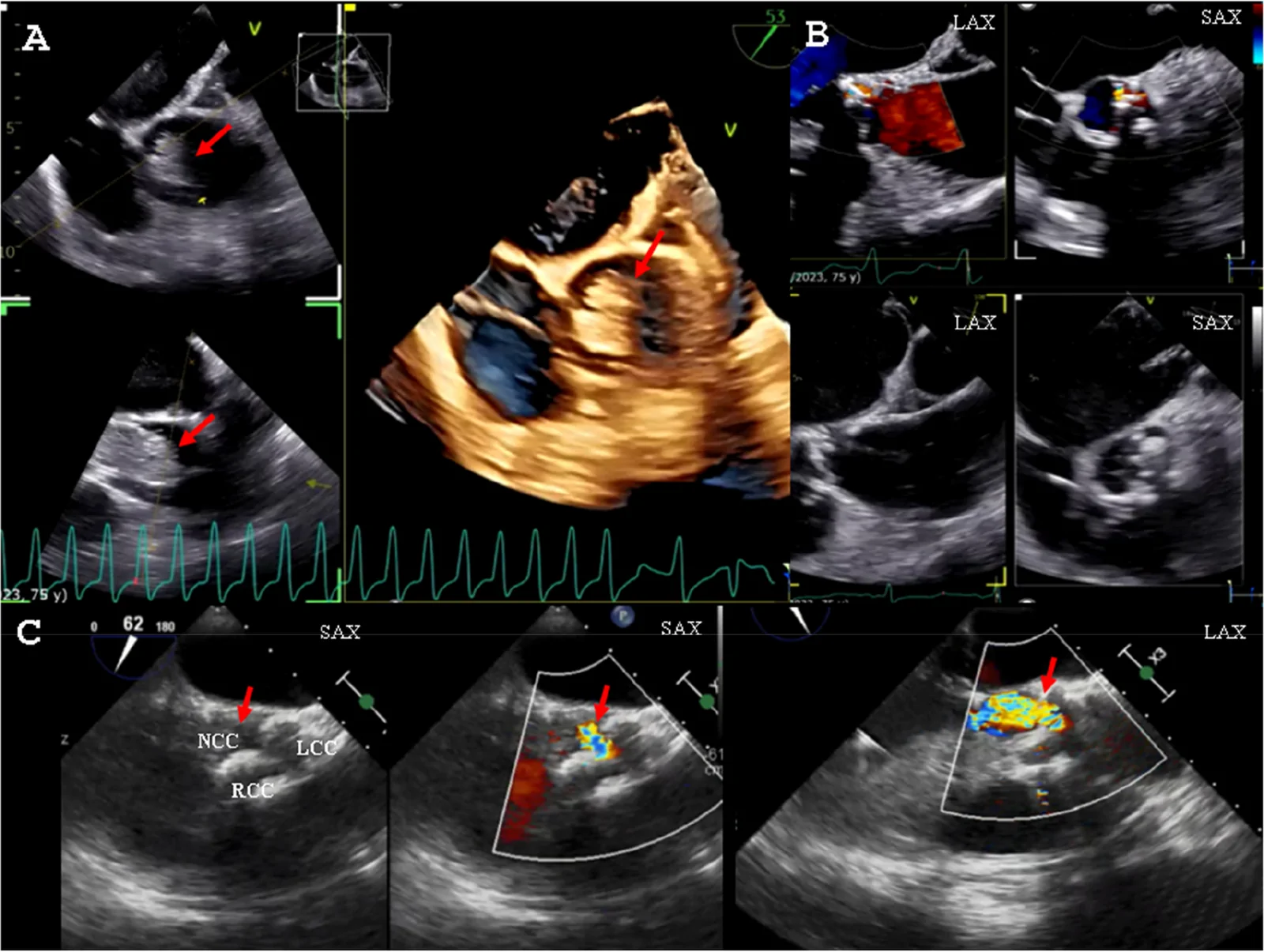
Balloon aortic valvuloplasty (BAV) in transcatheter aortic valve implantation (TAVI). **A** Real-time mini-transesophageal echocardiography (TEE) imaging of a severely calcified valve undergoing BAV. Red arrows point to the expanded balloon in 3D multiview (short axis [SAX], long axis [LAX], and 3D *en face* rendering views). **B** Mild aortic regurgitation (AR) was demonstrated post BAV. **C** In another example, acute severe AR occurred due to split noncoronary cusp (NCC) following BAV. Red arrows depict the split NCC commissure with resultant severe AR jet, that may cause acute hemodynamic compromise in patients with stiff and hypertrophied left ventricle. LCC, left coronary cusp; RCC, right coronary cusp

Prior to valve implantation, balloon aortic valvuloplasty (BAV) is frequently performed to increase cusps excursion and annular flexibility, thereby facilitating the delivery of the THV. Post BAV, imaging is vital to evaluate the hemodynamic impact of the dilation and to immediately identify life-threatening complications, such as acute AR from fracturing calcified leaflets, coronary artery occlusion (from distal embolization of calcified nodule), or cardiac tamponade. In this regard, intraprocedural TEE monitoring is the preferred modality as prompt assessment of the cause of hypotension or hemodynamic instability can be ascertained, alongside fluoroscopy uninterrupted (Fig. 9).

**Fig. 10:**
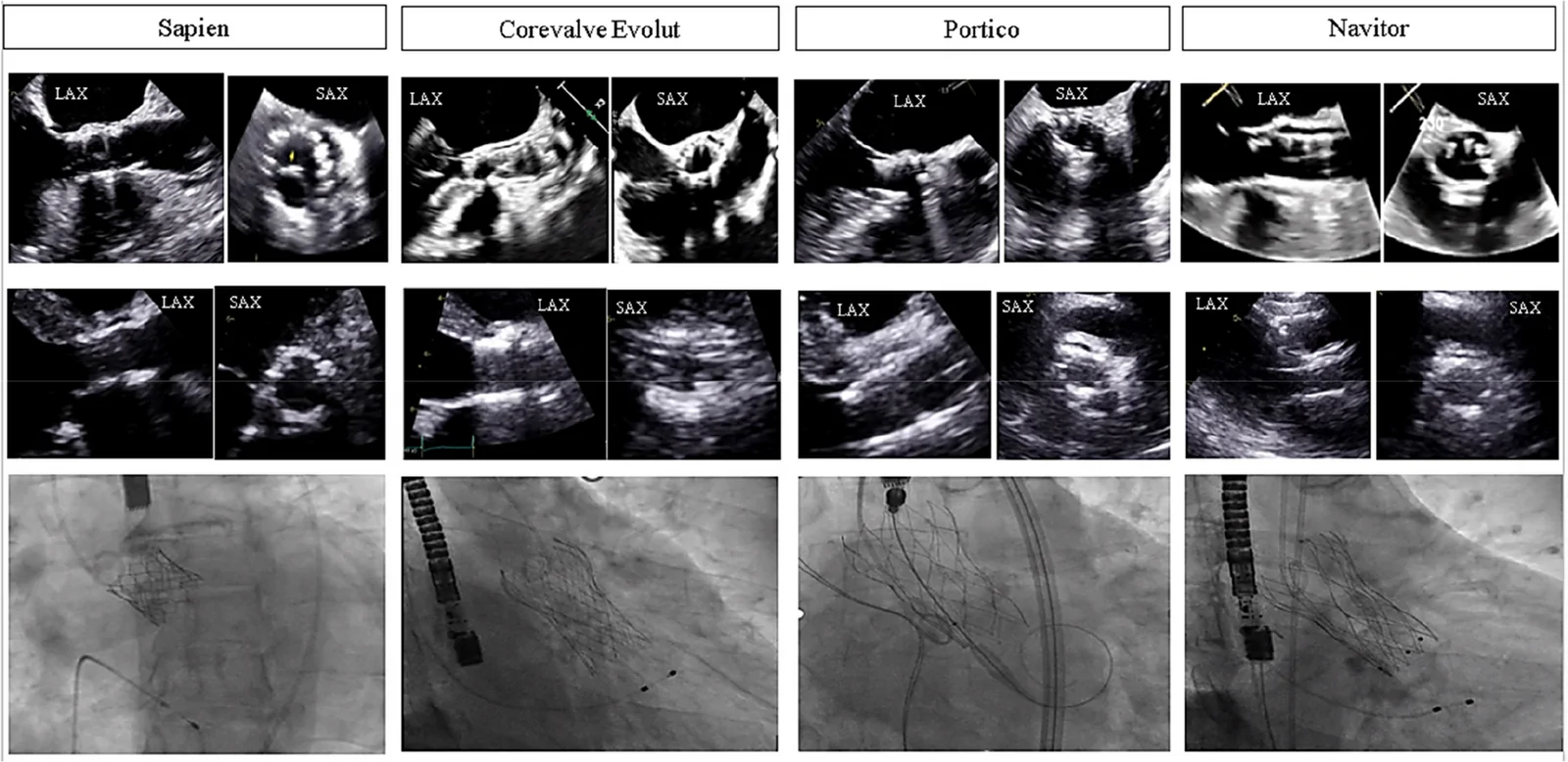
Echocardiographic and fluoroscopic appearance of the various transcatheter aortic valve implantation (TAVI) valves. Examples of commercially available TAVI valves (from balloon-expandable to self-expanding platforms) with normal valve function. First row, transesophageal echocardiography imaging; middle row, transthoracic echocardiography imaging; bottom row, fluoroscopy. LAX, long-axis; SAX, short-axis

While fluoroscopy remains the primary modality for valve deployment, echocardiography serves as a useful adjunct to ensure precise THV positioning. Because the deployment mechanisms differ between various TAVI platforms, it is critically important that imagers are familiar with the specific TAVI valves and their respective landmarks and requirements during deployment (Fig. 10). For self-expanding system (CoreValve, Medtronic; or Portico/Navitor valve, Abbott), it is recommended to confirm the proximal THV edge is at least 4 mm (but < 10 mm) below the annulus on the long-axis view of TTE or TEE (best on simultaneous long- and short-axis biplane imaging), to look for any paravalvular AR prior to full deployment since the self-expanding system is retrievable and repositionable if the initial position is unsatisfactory. For balloon-expandable valves (Edwards Lifesciences), the proximal part THV is generally recommended to be 5 mm below the annulus (with optimal final position at 2 mm below the annulus) as the valve will shorten upon full deployment from the ventricular end. In case the inflow portion of the THV cannot be imaged, the imagers should then focus on the distal edge of the THV which should cover the native leaflets, but remain below the sinotubular junction. Although TTE can be used for monitoring during valve deployment, TEE has the advantages of continuous monitoring, with a much better resolution and precision.

### Immediate after THV deployment

Following the deployment of the THV, either TTE or TEE may be utilized to comprehensively evaluate the THV positioning, shape, and leaflet motion, as well as its hemodynamic assessment, including the measurement of maximal velocity, mean pressure gradient, and calculation of the effective orifice area. Postdeployment assessment must prioritize the detection of both transvalvular and paravalvular AR (Figs. 1, 2), as moderate-to-severe postprocedural AR is strongly associated with increased short- and long-term mortality [28–31].

**Fig. 11:**
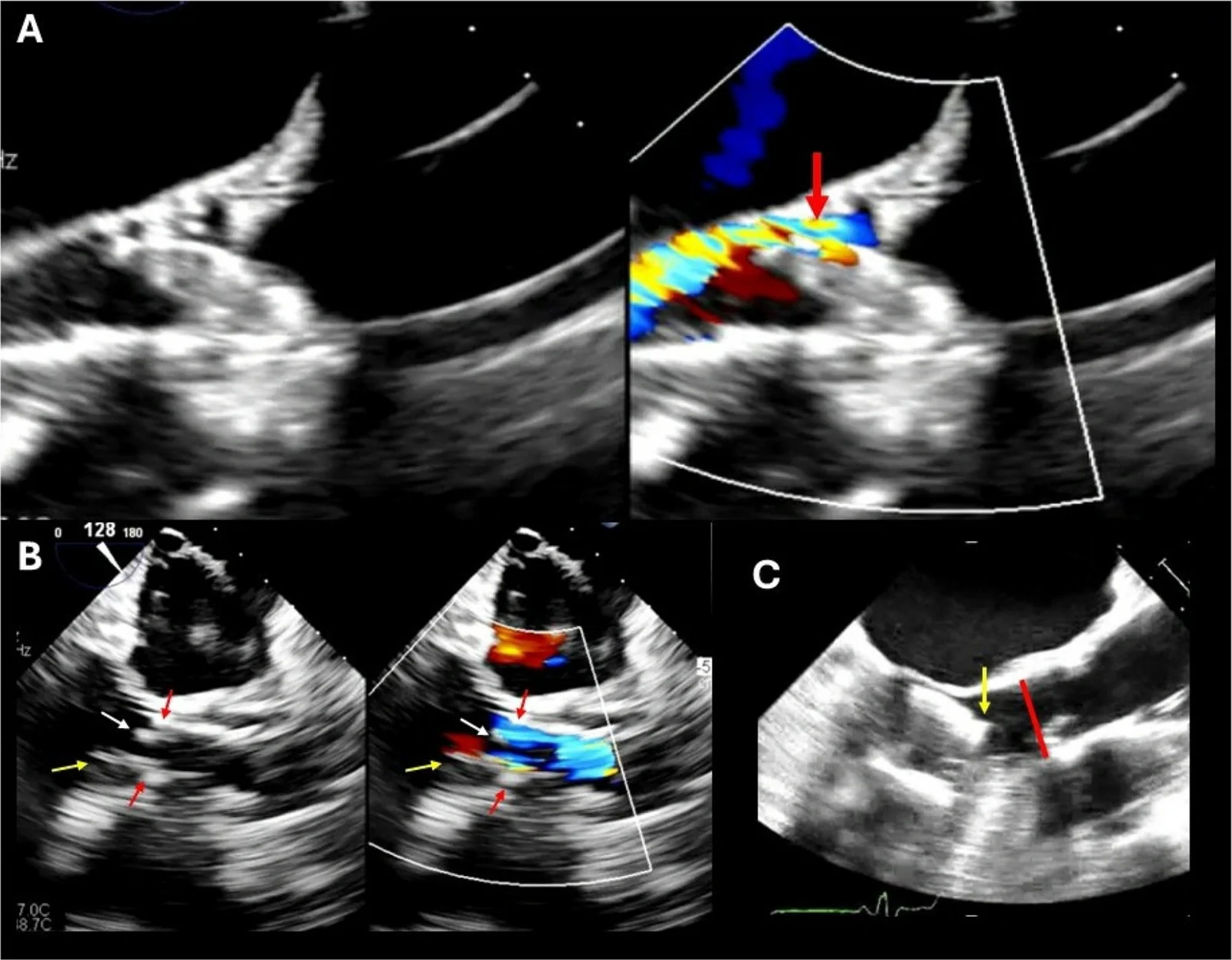
Underexpansion and malpositioning of transcatheter heart valve (THV). **A** Underexpanded THV (CoreValve, Medtronic) due to severe calcification causing moderate PVL (red arrow). **B** A case of aortic migration of the THV after following valve deployment with the final THV position above the annular plane. Native aortic leaflet (depicted in white arrow in midline) and the lower edge of the THV (depicted in red arrows) which is just above the native annulus. Guidewire in the left ventricle (LV) shown in yellow arrow. **C** Embolization of the THV into the LV (yellow arrow) in a patient with severely calcified and narrow aortic root after SAPIEN valve deployment. Red line denotes the annular plane

To overcome acoustic shadowing of the THV, it is crucial to image in multiple unconventional planes using multiwindow approach, to assess the entire circumference of the prosthetic to detect and localize any AR. While transvalvular AR is frequently a transient result of the stiff guidewire crossing the valve, which resolves upon wire removal, PVL presents a greater diagnostic challenge. Rarely, transvalvular AR may be due to leaflet damage (post balloon dilatation) or due to incomplete valve stent expansion which should be recognized promptly and addressed immediately (Fig. 2). Due to the complex and eccentric nature of PVL, the TEE or TTE probe must be rotated from medial to lateral in the long-axis view, supplemented by short-axis views at the annular level, preferably with simultaneous biplane imaging. A multiwindow approach, including deep gastric views (for TEE) or apical three- and five-chamber views (for TTE) can assist in jet detection, although they are often unreliable for quantifying jet area or length [32, 33]. According to the current guidelines, PVL is graded by its circumferential extent: less than 10% is considered mild, 10% to 29% is moderate, and greater than or equal to 30% is severe (Fig. 1) [12, 33]. However, because PVL is often characterized by multiple eccentric jets, determining its total regurgitant flow remains an area of uncertainty. Using 3D MPR, the 3D vena contracta area of the paravalvular jet in the short-axis view is an acceptable method to assess its severity that allows for summation of all the vena contracta areas in case of multiple jets (Fig. 1). The three primary factors contributing to post-TAVI PVL are prosthesis undersizing relative to the annulus, the severity and distribution of native AV calcification, and suboptimal prosthesis positioning [34]. In case of THV underexpansion due to severe calcification, additional balloon dilatation is required (Fig. 11A). When a THV is deployed too high or too low, the valve skirt fails to create an adequate seal, implantation of a second "valve-in-valve" prosthesis or “bailout surgery” is indicated (Fig. 11B, C). Therefore, immediate use of TTE or TEE imaging is critical for rapid detection and assessment of PVL severity and its underlying mechanism.

### Detecting complications during TAVI procedures

Intraprocedural TEE plays a pivotal role in TAVI procedures with high-risk anatomy as it offers continuous real-time monitoring, imaging guidance and rapid detection of complications.

### Cardiac tamponade and aortic dissection

While RV perforation is more commonly seen following the temporary pacing wire insertion during TAVI procedure, LV perforation can occur due to excessive wire manipulation in a small and hypertrophied LV cavity. Intraprocedural TEE provides real-time imaging, enabling early detection of complications such as pericardial effusion, cardiac tamponade or aortic dissection (Fig. 6). In the setting of tortuous iliofemoral anatomy, extensive vascular and aortic tortuosity [35], advancement of the catheter and sheath may require careful manipulation, to minimize the risk of vascular and aortic injury (Fig. 6C, D) [36].

### Bicuspid aortic valve

Bicuspid AV anatomy presents additional technical challenges, including asymmetric leaflet calcification and elliptical annular geometry that are associated with an increased risk of THV underexpansion to PVL, device malposition, conduction disturbance, coronary obstruction, and annular rupture [37, 38]. Although such patients were historically excluded from pivotal trials, contemporary data have suggested that, in carefully selected individuals with favorable anatomy, outcomes could approximate those of tricuspid AS [39, 40]. Due to huge variation in the morphology of bicuspid valves, CT is essential for preprocedural planning, consisting of in-depth analysis of AV complex and the aortic root at various levels (from level of LVOT up to the ascending aorta), using a “scroll techniques” to assess cusp morphology, calcium extent and distribution, raphe and coronary ostia location. Nonetheless, echocardiography (particularly TEE) during the TAVI procedure, is a valuable real-time modality to image AV complex during the entire procedure, from valve crossing to predilatation and valve implantation. Key is for close monitoring, immediate detection of complications and initiation of therapy to achieve optimal outcomes. Importantly post deployment, imaging the THV in short-axis view allows for immediate assessment of valve shape and leaflet motion, a crucial step in bicuspid valve to ensure that the valve is well positioned and fully expanded (Fig. 7). There is increasing evidence that THV underexpansion and eccentricity can impact valve hemodynamic, increasing the risk of leaflet thickening and clinical outcomes [41].

### Extensive, asymmetrical calcification of AV and aortic root

Extensive, bulky, and asymmetric calcification affecting the leaflets, sinotubular junction, LVOT, or annulus increases the likelihood of complications, including aortic root injury or annular rupture, moderate or severe PVL, incomplete prosthesis expansion, stroke and the need for permanent pacemaker implantation, and further maneuverers such as repeat balloon dilatation or a second valve (in case of valve migration or severe PVL). Intraprocedural TEE enables real-time visualization of calcium displacement or protrusion during balloon valvuloplasty and valve deployment, allowing prompt communication with the interventional team to anticipate potential serious complications (Figs. 9, 11). A meticulous examination of the aortic root and ascending aorta is required to detect signs of periaortic hematoma, dissection, or rupture, all of which can lead to life-threatening hemorrhage and cardiac tamponade. In cases of significant sinotubular junction calcification, optimal implantation depth may require positioning the valve just below the calcified segment. TEE is particularly advantageous in this setting, as it provides superior visualization for real-time imaging and exclusion of aortic pathology that extend beyond the proximal ascending aorta (Fig. 6C, D).

### Small hypertrophic LV and basal septal hypertrophy

Transient significant MR can be the consequence of guidewire entrapment in the mitral valve apparatus causing MR, particularly in patients with small LV cavity. Prompt identification with real-time intraprocedural TEE monitoring can help to alert the operators (Fig. 8A). Although uncommon, severe MR can occur as a result of leaflet perforation or chordal rupture during valve delivery or injudicious wire removal. TEE is particularly useful for assessing guidewire entanglement and identifying dynamic causes of MR, such as postdeployment LVOT obstruction with systolic anterior motion in patients with basal septal hypertrophy (Fig. 8B). Rarely, THV embolization (“popping up”) may occur in patients with severe basal septal hypertrophy receiving balloon-expandable valve due to nonspherical landing zone rendering it unstable to seat the THV [42]. Diastolic MR may also occur in the setting of complete heart block post TAVI, therefore understanding the precise mechanisms of MR on TEE is key for counter measures to address this complication.

### Severe LV and or RV impairment

In patients with preexisting severe LV and/or RV impairment represent an extreme high-risk group who are sensitive to changes in loading condition, as well as changes in hemodynamic. Intraprocedural monitoring with uninterrupted TEE imaging can help to detect new regional wall motion abnormalities or valvular regurgitation and explain causes of hypotension in such patients. Should any new ventricular dysfunction be identified, a root aortogram or selective coronary angiography must be performed immediately to confirm the diagnosis and intervene as appropriate. Rarely, acute LV stunning may occur following BAV alone in patients with severely impaired LV function, albeit no new AR due to poor LV reserve. Therefore, prompt exclusion of major complications using real-time echocardiographic assessment is key in case of hemodynamic compromise.

## Role of ICE imaging in TAVI

ICE imaging is an attractive alternative to TEE or TTE imaging as it provides real-time high-resolution images due to close proximity to the cardiac structures imaged during structural heart interventions and can be performed under LACS, without the need for GA, by the same operator. Moreover, it is capable of continuous, rather than intermittent procedural guidance and monitoring. Previous experience had reported a lower need for probe repositioning (0.1 ± 0.3 maneuvers vs. 5.7 ± 0.7 maneuvers, P < 0.001) during ICE-guided compared to TEE-guided TAVI procedures [43]. Currently, ICE imaging is commercially available in 8 F catheter (for 2D ICE) or 9–10F catheter (for 3D ICE) depending on the systems used [44, 45]. For TAVI procedures, the steerable ICE catheter is introduced via either the right common femoral or right internal jugular vein, advanced into right atrium, and finally to the superior cavoatrial junction [44]. The main ICE view during TAVI imaging is the longitudinal view from the cavoatrial junction, obtained with the catheter tip tilted towards the ascending aorta, in counterclockwise rotation. This view enables good preprocedural imaging of the LVOT, native AV morphology and aortic root. During the procedure, this is the view that is maintained throughout the procedure as it permits visualization of catheter placement and positioning of the THV mounted on delivery catheter. After THV deployment, the short-axis view with the ICE catheter in right atrium, and its tip tilted by 90° towards aortic annulus, is used to assess for PVL. Finally, the ICE catheter is advanced into RV across the tricuspid valve to obtain the long-axis view of the LV, similar to parasternal long-axis view on TTE, which is helpful in assessing the LV ejection fraction and to look for any pericardial effusion. Additional information about MR and tricuspid regurgitation can also be obtained. Thus, real-time imaging with ICE during TAVI procedure helps in the assessment of THV position, implantation depth, and evaluation of PVL severity after deployment, as well as for immediate detection of intra and postprocedural complications [44].

Recently, ICE guidance under LACS was shown to be comparable to TEE guidance under GA in a propensity matched cohort of patients undergoing TAVI in terms of safety and clinical efficacy, with similar incidences of moderate-to-severe PVL, new permanent pacemaker implantation and major bleeding [46]. Therefore, ICE guidance is a safe alternative to TEE imaging during TAVI procedures. Moreover, transjugular ICE guidance has also been shown to reduce the risk of conduction disturbances/implantation of permanent pacemaker with real-time direct visualization of the membranous septum and achieving a high position of the THV using self-expanding valves [47]. With 3D ICE, evaluation of the entire aortic root and AV anatomy is superior to conventional 2D ICE as it offers 3D based annular sizing and simultaneous biplane imaging during TAVI, although the 3D volume rendered achieved with 3D ICE is smaller than that of conventional 3D TEE [44, 45].Another advantage of ICE is shown by Bartel et al. [48], who reported a reduction in the average amount of contrast use with primarily ICE guidance to 20.1 ± 8.7 mL, from 72.0 ± 30.7 mL with fluoroscopy-guided TAVI. Therefore, a tailored strategy integrating ICE and fluoroscopy might be potentially beneficial in patients with chronic kidney disease. In addition, ICE provides reliable intraprocedural pressure gradients assessment that is comparable to preprocedural TEE [43]. In summary, ICE may be particularly helpful in high-risk patients undergoing TAVI with challenging TTE windows, or in those with contraindications for TEE, such as esophageal stricture, ulceration or varices.

Despite these advantages, ICE is not frequently used in TAVI as ICE catheters are expensive and are limited to a single use. However, this cost must be balanced with the additional imaging information that is required during complex procedures to help guide and/or detect potentially life-threatening complications. Although the use of ICE imaging is generally safe, the incidence of serious complications (vascular injury or cardiac tamponade) is reported to be 1%–2% [49]. Moreover, the ICE operator has to be careful during manipulation as it may risk interference and dislodgement of pacing wires in patients with cardiac implantable device. In addition, there is a learning curve for familiarization with ICE catheter manipulation to obtain optimal views. The cost-effectiveness of ICE for guiding TAVI procedure will require future research, although it is likely to be a patient-centered approach balancing the risks and benefits in each TAVI candidate.

## Conclusions

**Fig. 12:**
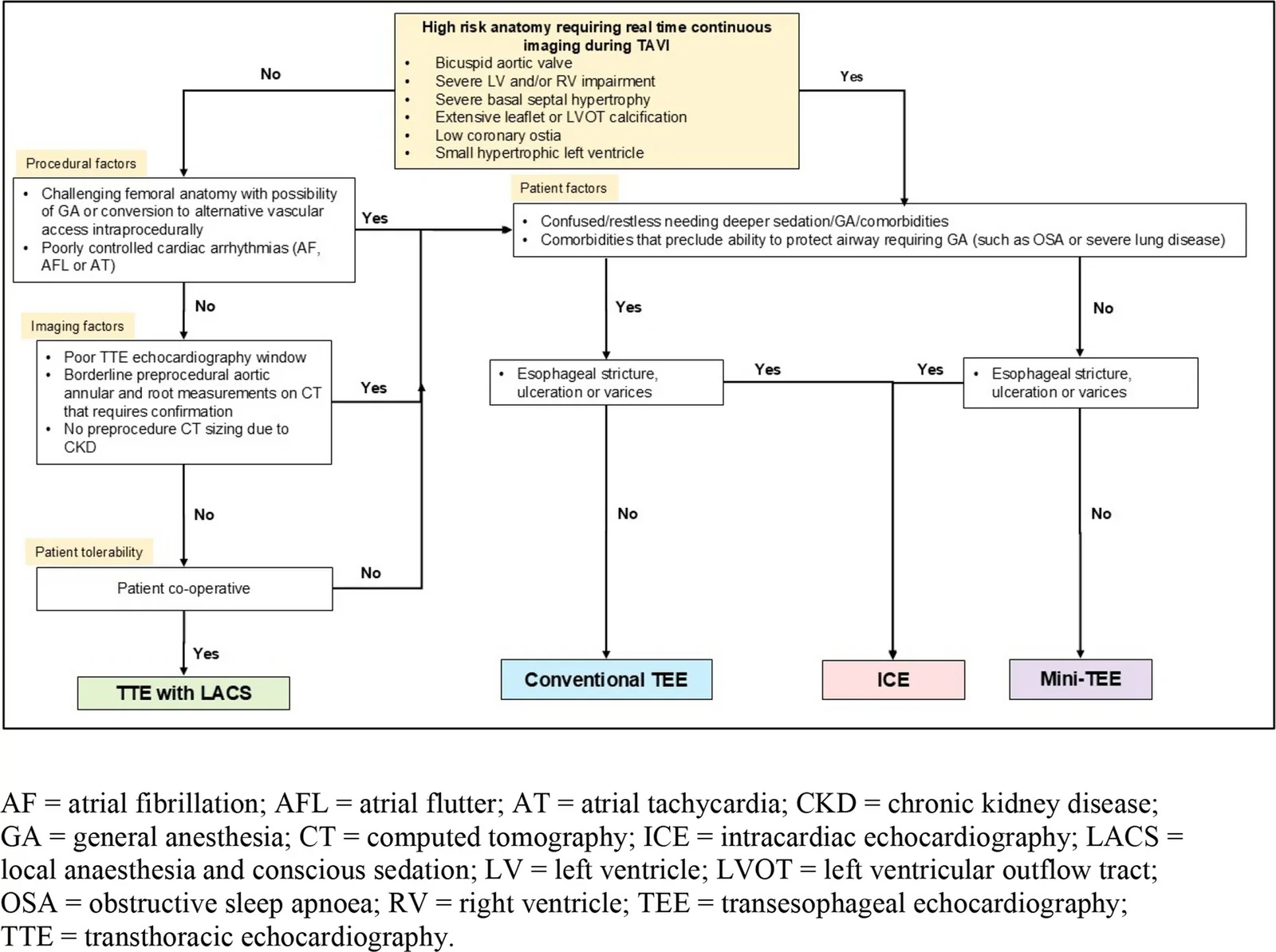
Practical decision pathway for use of echocardiography modality during transcatheter aortic valve implantation (TAVI). AF, atrial fibrillation; AFL, atrial flutter; AT, atrial tachycardia; CKD, chronic kidney disease; GA, general anesthesia; CT, computed tomography; ICE, intracardiac echocardiography; LACS, local anesthesia and conscious sedation; LV, left ventricle; LVOT, left ventricular outflow tract; OSA, obstructive sleep apnea; RV, right ventricle; TEE, transesophageal echocardiography; TTE, transthoracic echocardiography

Despite technological advancements and operator experience, complications associated with TAVI remain a significant clinical concern. Echocardiography plays a central role in before, during, and after TAVI deployment as it offers real-time monitoring, imaging guidance, and rapid detection of complications. The decision to employ a time efficient, limited imaging protocol versus comprehensive, continuous monitoring should be tailored to patient-specific anatomy and the anticipated risk of complications (Fig. 12). While minimalist approaches are feasible, TEE remains valuable in high-risk anatomy or complex cases. Through meticulous patient screening, and individualized imaging protocol, and the synergy of operator expertise with evolving transcatheter technology, TAVI is poised to become an even safer procedure.

## Data Availability

No datasets were generated or analysed during the current study.

## Abbreviations

AR: Aortic regurgitation
AS: Aortic stenosis
AV: Aortic valve
BAV: Balloon aortic valvuloplasty
CT: Computed tomography
GA: General anesthesia
ICE: Intracardiac echocardiography
LACS: Local anesthesia and conscious sedation
LV: Left ventricular
LVOT: Left ventricular outflow tract
MPR: Multiplanar reconstruction
MR: Mitral regurgitation
PVL: Paravalvular leak
RV: Right ventricular
TAVI: Transcatheter aortic valve implantation
TEE: Transesophageal echocardiography
THV: Transcatheter heart valve
TTE: Transthoracic echocardiography
